# Strain Measurement with Optic Fibers for Structural Health Monitoring of Woven Composites: Comparison with Strain Gauges and Digital Image Correlation Measurements

**DOI:** 10.3390/s23249794

**Published:** 2023-12-13

**Authors:** Carlo Boursier Niutta, Andrea Tridello, Raffaele Ciardiello, Davide S. Paolino

**Affiliations:** Department of Mechanical and Aerospace Engineering, Politecnico di Torino, 10129 Turin, Italy; andrea.tridello@polito.it (A.T.); raffaele.ciardiello@polito.it (R.C.)

**Keywords:** optic fiber, strain gauge, Digital Image Correlation, woven composites

## Abstract

In this work, the strains measured with optic fibers and recorded during tensile tests performed on carbon/epoxy composite specimens were compared to those recorded by strain gauges and by Digital Image Correlation (DIC). The work aims at investigating the sensitivity of embedded and glued optic sensors for structural health monitoring applications in comparison with strain gauges and the full field strain map of the DIC. Acrylate, polyimide optic fibers, and three strain gauge sizes are considered to compare the three techniques. Results show hard polyimide-coated sensors are more sensitive to the material pattern than soft acrylate-coated fibers, which also require extensive adhesion length. The work shows a comparable size of strain gauges and material meso-structure is also critical for properly assessing material properties. The Young’s modulus computed with the three different techniques is used to define a strategy that supports the selection and the proper size of the adopted strain measuring system for structural health monitoring of composite materials.

## 1. Introduction

Real-time structural health monitoring for damage assessment has a key role in the reliability of composite materials for in-service structures. Composite materials are known for their superior capability to withstand damage with respect to standard metals. However, several types of damage mechanisms, which can interact and whose evolution is still difficult to predict, affect composite materials [1]. Furthermore, damage is usually localized, i.e., it affects only a local portion of the material, especially in the early phases of its evolution [2,3]. Therefore, techniques for structural health monitoring must be particularly sensitive to local material variations.

Optic fibers are regarded as a promising solution for the structural health monitoring of composites [4]. Among the most favorable factors we can mention: (i) by measuring the deformation, optic sensors provide quantitative information on the health state of the component [5]; (ii) optic sensors based on the backscattering phenomenon have the highest spatial resolution (even smaller than 1 mm) [6,7], which allows obtaining an almost continuous map of the deformations within the component; (iii) thanks to their small size, optic sensors can be embedded within the composite structure. Differently from the Fiber Bragg–Gratings (FBG) sensors, which provide information only in specific spots called grating, by local alterations of the optic fiber, the so-called Distributed Fiber Optic Sensors (DFOS) exploit the internal fiber defectivity to track the deformation, thus allowing to consistently increase the spatial resolution. For this reason, the present study has considered DFOS based on the Rayleigh backscattering phenomenon.

The use of optic sensors for structural health monitoring, however, presents several challenges related to the manufacturing process, to the local alteration induced by the optic fiber and to the influence of the sensor characteristics on the acquired signal. The manufacturing process of the composite structure with embedded optic fibers presents several complexities which depend on the use of pre-pregs or on resin-infused processes. In the literature, both pre-pregs and resin-infused processes have been investigated. In [8], the authors have shown a possible strategy for embedding optic sensors in a glass-fibers composite vacuum-infused by epoxy resin. To access the fiber extremities after the infusion process, the optic sensor passed through the vacuum bag, later sealed. However, the curing cycle might induce stresses that could break the optic sensor in correspondence of the exit spot. Rufai et al. [9] and Kinet et al. [10] therefore suggested the use of a thin Teflon film in correspondence of the exit spot. Other solutions aim at protecting the optic sensor by embedding a massive connector, which however can weaken the composite material and induce delamination [11]. More recently, despite the inherent complexities, the so-called free-spacing coupling technique, which exploited a large core fiber welded to the proper optical sensor and a small breach in the material, has been proposed [12].

The embedment of optic fibers is also critical as the fibers are prone to break when manipulated. Optic fibers are usually coated with a polymeric coating which has two main functions: (i) the rapid suppression of high transverse modes for a clear light signal; (ii) protecting the fiber and increasing its flexibility. Accordingly, the presence of the coating increases the whole optic sensor diameter, thus inducing a local alteration of the material microstructure with consequences on the mechanical strength [11,13,14,15]. Optic fibers indeed present a central core, usually of 50–60 µm diameter, where the light travels, a cladding of 125 µm diameter, which usually has a smaller refractive index to confine the light in the core, and the coating which leads the final diameter to 150–250 µm. The coating refractive index is also fundamental for the attenuation of the signal. In particular, if the coating has a refractive index smaller than that of the cladding, the light signal is easily reflected to the core, thus reducing the so-called micro-bending losses [16]. On the contrary, when the refractive index of the coating is higher than that of the cladding, the light signals can pass through the cladding and be confined within the coating, thus resulting in signal losses. By bending the optic sensor, the losses increase, which can be also critical for the embedment of the optic sensor. The coating is also fundamental for transferring by shear the deformation of the structure to the optic fiber [17,18]. Material properties and thickness of the coating indeed affect the shear stiffness. The coating elasticity attenuates the deformation of the inner core, thus affecting the measurement [7,19].

These considerations are particularly important in the case of carbon fiber woven composite, which are inhomogeneous materials even at the mesoscale of the woven fabric. The material inhomogeneity demands for accurate information at the texture mesoscale to allow the identification of proper strategies for health state monitoring of components made of carbon fiber woven composite. Furthermore, the embedment of optic fibers within the composite is particularly challenging, given the woven texture.

In this paper, we investigated the strain measurements of embedded optic fibers in the elastic field of a carbon fiber woven composite. Optic fibers with soft, i.e., acrylate, and hard, i.e., polyimide, coatings have been considered. Optic sensors have been either embedded within or glued on the specimens to compare the strain results and the manufacturability of the coatings. In this regard, different engagement lengths of the optic sensors have been also compared. Results have been compared to Digital Image Correlation (DIC) acquisitions and to standard strain measurement with strain gauges characterized by different dimensions, which were reference techniques for strain acquisition. The comparison has been addressed in terms of the elastic modulus of the material determined through the three strain acquisition systems, to provide a unique value which allowed facilitating the discrimination between the three techniques, while providing consistent mechanical information. Given the inhomogeneous meso-structure of the material, resulting from the longitudinal and transverse yarns, deformations caused by the application of the mechanical load were also inhomogeneous, which demanded high accuracy in strain acquisition for health state monitoring.

## 2. Materials and Methods

In this section, the carbon fiber woven fabric composite is first described. The specimen preparation with embedded optic fibers is then addressed. Finally, details of the tensile tests with the different strain acquisition methods are reported.

### 2.1. Material and Specimen Preparation

Carbon fiber pre-pregs, namely XC110 (XPREG, Stoke-on-Trent, UK), have been used for the specimen preparation. The pre-pregs present a 2 × 2 twill weave with a tow wavelength of 8 mm and are impregnated with epoxy resin. The areal weight is 416 g with 6000 fiber filaments per tow both in the warp and in the weft direction. Each lamina has a consolidated thickness of 0.44 mm and a nominal fiber volume fraction of 55%. The tested specimens have been produced with 4 layers all aligned with the loading direction, i.e., the stacking sequence was [0]_4_.

**Figure 1:**
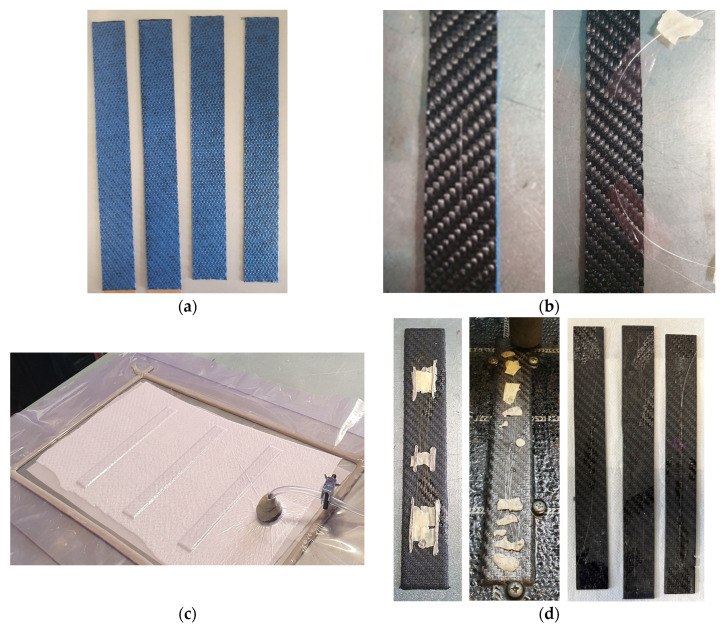
Specimen preparation: (**a**) hand-cut pre-pregs strips; (**b**) optic fiber threading through two plies, optic fiber tensioning and release film interposing to avoid adhesion; (**c**) vacuum-pack for cure cycle; (**d**) waterjet cutting after curing cycle and final specimens.

Figure 1 shows the main steps followed for the preparation of the specimen with embedded optic fibers.

The specimen preparation consists of four main steps: (i) hand-cutting strips of the prep-pregs; (ii) embedding the optic sensor; (iii) vacuum-bagging the material and curing process; (iv) waterjet cutting to the final shape. More in detail, for each specimen, four strips were cut from the material roll, as shown in Figure 1a. As the goal was to embed the optic fiber in the middle of the laminate, that was between the second and third layers, the optic fiber was first threaded through two layers and then the two layers embedding the optic fiber were disposed over the other two layers, as shown in Figure 1b. The optic fiber was tensiled to avoid internal waviness and guarantee the optic fiber alignment with the specimen. The optic fiber flaps coming off the specimen were also covered with releasing wax and a release paper film was interposed between the optic fiber and the pre-pregs, as shown in Figure 1b, to avoid the adhesion of the optic fiber to the specimen during the cure cycle. The specimens were thereafter disposed on a glass coated with releasing wax to avoid adhesion, covered with a peel-ply and a breather ply to facilitate the removal of entrapped air, and vacuum-packed by means of a bag and a sealant tape (Figure 1c).

The material was then cured under vacuum. The cure cycle lasted 15 h and consisted of a first ramp up to 70 °C with a ramp rate of 1 °C/min. After 4 h at 70 °C, the temperature was further increased up to 85 °C which was held for 10 h. The natural cool-down in the oven completed the cure cycle.

Once the material was cured, 200 mm × 25 mm specimens were obtained by cutting with a water jet machine, paying attention to avoid damaging the optic fibers (Figure 1d). In in the case of the long embedded optic fibers, to avoid possible damage, the extremities of the material were not cut and only the width was adjusted to 25 mm through the water jet machine, thus guaranteeing a uniform cross-section along the specimen length. It is worth remarking the extremities of the specimen are engaged in the testing machine grips and therefore do not participate in the specimen deformation.

### 2.2. Mechanical Tests

The carbon fiber woven specimens were subjected to quasi-static tensile tests on a servo-hydraulic Instron 8801 machine. The displacement rate was 2 mm/min. The maximum force has been limited to 5 kN, which corresponded to about 120 MPa, to investigate the elastic field of the material while avoiding damaging the specimens. According to the manufacturer datasheet, the tensile strength of the retained composite is 520 MPa. The elastic modulus of the investigated material assessed through the three strain acquisition systems was compared. In particular, the Young’s modulus was calculated by firstly computing the stress from the applied tensile load and the specimen cross-section and then by linearly interpolating the strain measurements and the resulting stress with the least squares method. As the tests were performed in the elastic field of the material, all the acquired values were considered for the linear interpolation. Therefore, according to the least squares method, the Young’s modulus was determined as:

(1) $$E=\frac{\sum_{i=1}^{n}\left(\varepsilon_{i}-\bar{\varepsilon }\right)\left(\sigma_{i}-\bar{\sigma }\right)}{\sum_{i=1}^{n}{\left(\varepsilon_{i}-\bar{\varepsilon }\right)}^{2}}$$

where $\varepsilon_{i}$ and $\sigma_{i}$ are the *i*-th strain and stress values, respectively, $\bar{\varepsilon }$ and $\bar{\sigma }$ the average value of the acquired strain and stress, respectively, and n is the total number of acquired values.

Table 1 summarizes the investigated strain acquisition systems and the related addressed aspects.

Regarding the optic fiber, this study investigated the influence of the location of the optic fiber, i.e., glued on the outer surface of the specimen or embedded within the laminate, the influence of the coating material, i.e., acrylate coated fiber and polyimide-coated fiber, and the influence of the fiber length glued or embedded in the specimen. Regarding the length, long sensors were engaged for 100 mm, while short sensors were engaged for 50 mm. A pigtail, at one side, and a coreless terminal fiber, namely a FG125LA by Thorlabs (Newton, NJ, USA) [20], at the other side, were micro-welded to the fiber optic sensors with a Fujikura 90S+ system.

Two types of coating have been considered: a polyimide coating, fiber name SM1550P by Thorlabs [21], and a double-layer acrylate coating, fiber name G.657.A1 by Optokon (Jihlava, Czech Republic). The polyimide is thinner and more rigid with respect to the acrylate coating and therefore more suitable for mechanical applications [17]. On the contrary, given its high flexibility, the acrylate coating is easier to handle and therefore to embed within a laminate, being less sensitive to the deformation induced by the curing process. According to [16], the Young’s modulus of the double-layer acrylate coating is 2.0 GPa, while that of the polyimide coating is 4.9 GPa. Table 2 reports the main properties of the retained optic fibers.

The optic fiber interrogator was a LUNA OdiSi system (LUNA, Roanoke, VA, USA) that is based on the Rayleigh backscattering effect. The presence of impurities reflects the light travelling along the fiber, thus allowing a continuous measurement of the strain. More in detail, the LUNA system generates a swept light signal of the light and detects, in the frequency domain, its changes due to the applied load and the change in the distance between the impurities. In the tested specimens, the strain spatial resolution was set at 0.65 mm.

Regarding the strain gauges, three strain gauges with increasing grid length were considered, corresponding to 0.3 mm, 3 mm, and 6 mm. The strain gauge with grid length of 0.3 mm was an HBM 1-LY11-0.3/120. The strain gauge with grid length of 3 mm was an HBM 1-LY48-3/350, whereas the strain gauge with grid length of 6 mm was HBM 1-LY48-6/350. The National Instruments NI 9944 (for the strain gauges with the grid length of 0.3 mm) and NI 9945 (for the strain gauges with grid lengths of 3 mm and 6 mm) were used for the quarter bridge completion of the Wheatstone bridge. The strain gauge signals were finally acquired with the NI 779521-01 device acquisition system at an acquisition frequency of 1.6 kHz.

**Figure 2:**
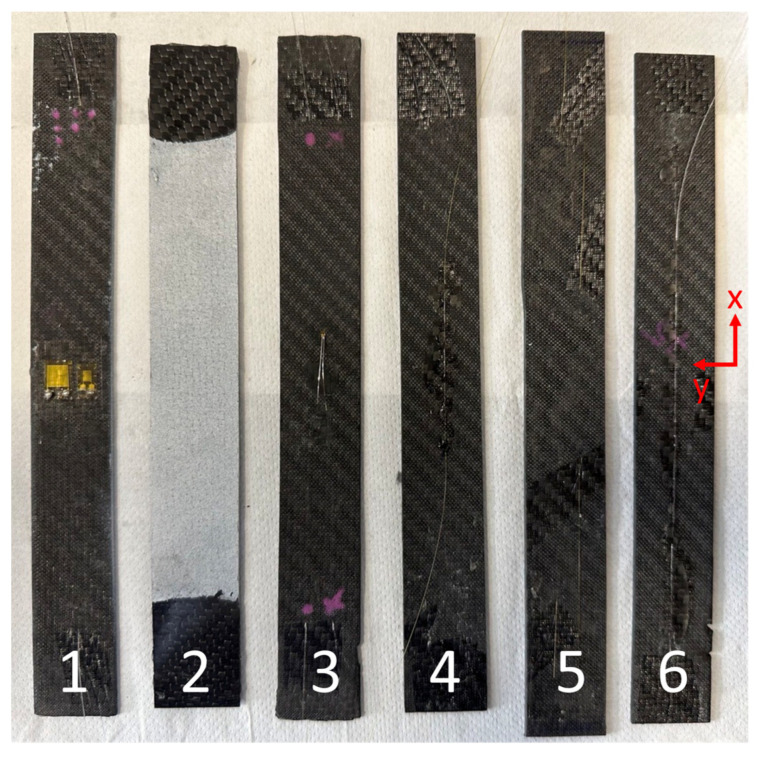
Tested specimens with black-on-white speckle for DIC, embedded and glued optic fibers and strain gauges of different sizes.

For the DIC system, the tests were recorded with two 8.9 MP cameras to obtain the full three-dimensional displacement and strain map fields. A black-on-white speckle pattern was airbrushed on the specimens, as shown in Figure 2. The recorded images were analyzed with VIC 3D 9.1.6 software, considering a subset size of 36 px and a step size of 6 px, thus guaranteeing a ratio between the subset size and step size smaller than 1/3 as recommended in [22]. The resulting spatial resolution was equal to about 0.11 mm. To calculate the reference Young’s modulus, for each instant of the test, the strain field was averaged over the whole framed area. Furthermore, to investigate the influence of the averaging area, i.e., the area over which the average of the measured strains was computed, on the determined elastic property, its size was varied and randomly disposed within the framed area.

Figure 2 shows the tested specimens, where the optic fibers, both embedded and glued on the specimen surface, the speckled specimen for the DIC and the specimens with strain gauges of different sizes can be recognized.

The axial loading direction corresponds to the x-axis, while the transverse direction corresponds to the y-axis. Although the presence of embedded optic sensors determines a local alteration of the woven fabric, previous studies [11,13,14,15] show its influence on the elastic behavior of the material is negligible. Therefore, discrepancies between the specimen properties are assumed limited and negligible and only due to non-controllable factors. For the sake of clarity, the strains of specimen #1 were measured through the DIC, by speckling the opposite surface of that shown in Figure 2, through the strain gauges of dimensions 3 mm and 6 mm and an acrylate sensor embedded in the middle of the laminate for a length of approximately 100 mm (long sensor). One surface of specimen #2 was speckled for DIC acquisition, while an acrylate sensor was embedded in the middle of the laminate for a length of approximately 50 mm (short sensor) and came out from the opposite surface. The strain gauge of 0.3 mm was mounted on specimen #3, which also contained an acrylate long sensor. A polyimide sensor was embedded in specimen #4, although it did not work properly. The strains of this specimen were measured through a polyimide sensor glued on the surface, as shown in Figure 2. In specimen #5, both long and short polyimide sensors were embedded, although only the short sensor properly worked after the curing cycle. Finally, specimen #6 contained a long acrylate sensor, which however broke. The strains of this specimen were measured through an acrylate sensor, firstly glued for a length of 50 mm and then for a length of 100 mm.

## 3. Results

In this section, the results of the strain measurements and resulting Young’s modulus are analyzed for the three strain acquisition systems. Results are then compared and discussed. In the following figures, the x-coordinate corresponds to the longitudinal direction, i.e., the applied load direction.

### 3.1. Optic Fiber Results

**Figure 3:**
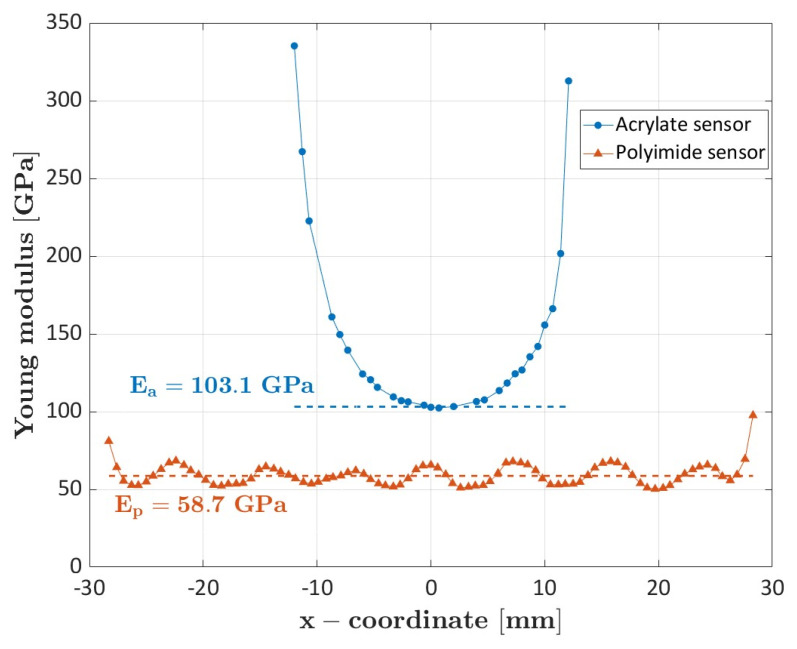
Comparison of the elastic modulus obtained from the polyimide and acrylate optic sensors glued on the specimen surface.

Figure 3 shows a comparison of the elastic modulus calculated with the optic fibers coated with acrylate and polyimide and glued on the specimen surface for a length of approximately 50 mm (specimen #4 and specimen #6).

The Young’s modulus calculated through the polyimide-coated sensor oscillates around a mean value of 58.7 GPa. The oscillations are caused by the mesoscale structure of the material with the alternate presence of longitudinal, i.e., stiff, and transverse, i.e., complaint, tows. The longitudinal tows can be indeed seen as a local unidirectional carbon fiber composite loaded along the fiber direction, while the transverse tows as a unidirectional carbon fiber composite loaded transversely with respect to the fiber direction [23]. The deformations accordingly oscillate, despite the homogenizing influence of the surrounding tows. The period of the oscillations captured by the polyimide sensor almost corresponded to the size of the woven tow. 

On the contrary, the Young’s modulus calculated from the strains acquired by the acrylate-coated optic fiber is consistently higher and nonconstant along the engaged length. The mean value computed by averaging the values comprised in the 2% with respect to the minimum was equal to 103.1 GPa. This discrepancy is due to the lower Young’s modulus and to the higher thickness of the acrylate coating compared to the polyimide coating. When the load is applied to the specimen, the coating transfers the deformation to the optic sensor by shear. The lower Young’s modulus combined with the higher thickness favors a significant shear deformation within the coating, which affects, in turn, the strain measurement and leads to the U-shaped trend [17]. More in detail, high values of the Young’s modulus of the coating favor the transfer of the specimen deformations to the optic fiber, while low values prevent the optic fiber from having the same deformation of the specimen. Similarly, in case of thin coating, the tangential stresses, and so the shear deformations, can be assumed constant within the coating, thus allowing the optic fiber to have the same deformation of the specimen. With thick coatings, the deformation of the specimen is only partially transferred to the optic fiber, i.e., the shear strains are not constant within the coating, thus resulting in less accurate results. Accordingly, the acrylate-coated fiber cannot capture strain variations due to the mesoscale structure of the material.

**Figure 4:**
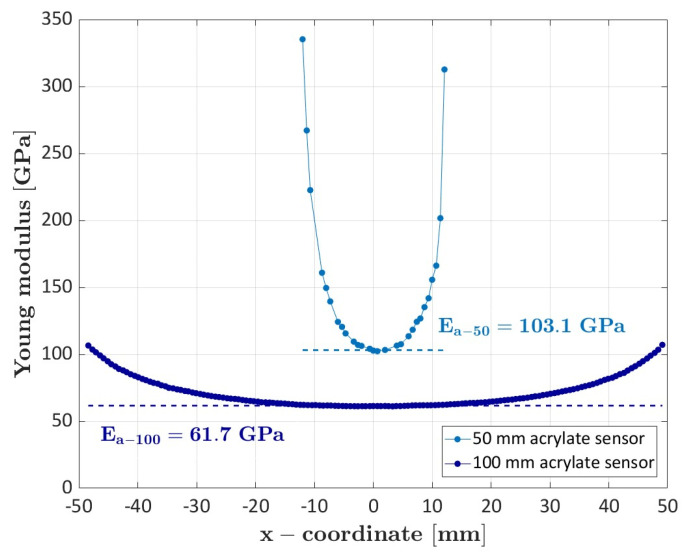
Comparison of the elastic modulus obtained from the acrylate optic sensors glued on the specimen for a length of 50 mm and 100 mm.

The significant influence of the shear deformation within the coating can be also shown by comparing the results obtained with the acrylate-coated fibers engaged for a length of 50 mm and 100 mm (specimen #6), reported in Figure 4.

By increasing the engaged length, it is possible to identify a region where the shear strains within the coating are very limited and the optic sensor deforms in accordance with the specimen. However, the acrylate optic fiber is still not able to capture the differences between the longitudinal and transverse tows. These results suggest acrylate-coated optic sensors are very sensitive to the engaged length. Furthermore, given the limited sensitivity to local material variations, like those due to local damage, the use of acrylate sensors in mechanical applications, e.g., for structural health monitoring, must be carefully evaluated. However, acrylate-coated sensors are easier to handle and to embed within a structure with respect to polyimide-coated sensors. In the specimen preparation, we discarded 2 of the 3 embedded polyimide sensors (long sensors in specimen #4 and in specimen #5), as the fibers broke during the curing cycle, or the light signal was too weak for proper strain measurement. On the contrary, the acrylate sensor was easily embedded and only one sensor, embedded in specimen #6, was discarded. Regarding the weak signal obtained in some embedded fibers, this is likely due to excessive micro-bending losses. Indeed, to thread the fibers through the material layers, the optic sensors are bent, which can cause a signal loss. This aspect further challenges the manufacturability of composite structures with embedded optic fibers and must be properly accounted for.

**Figure 5:**
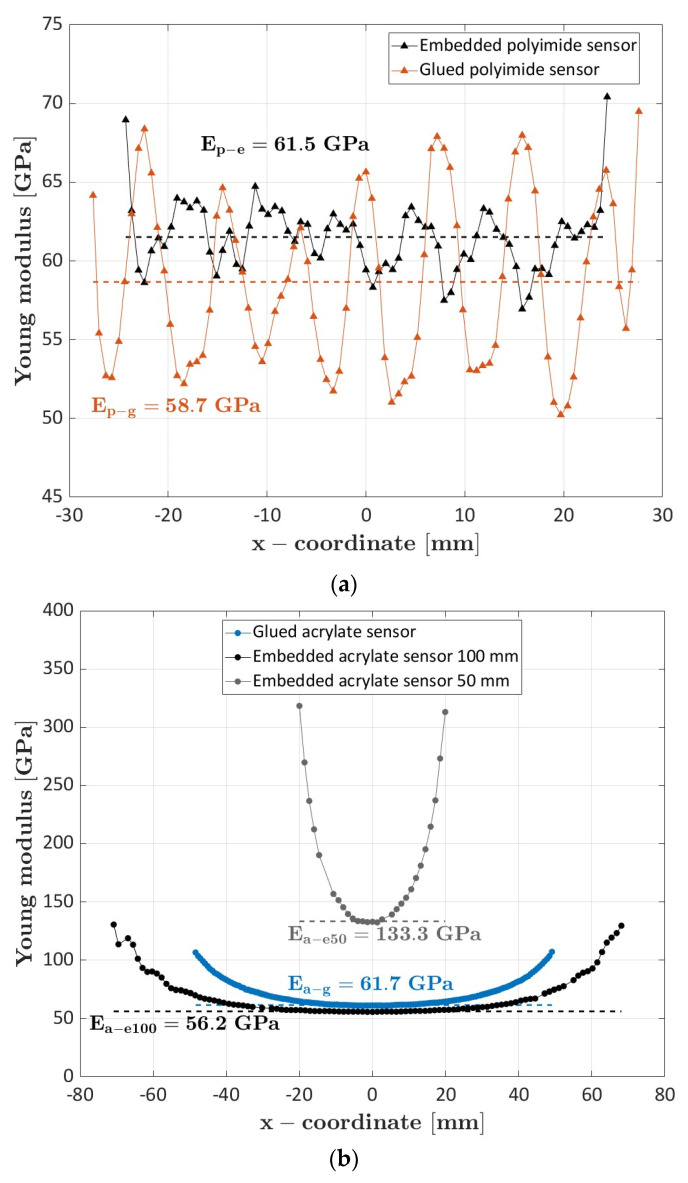
Comparison of the elastic modulus obtained from glued and embedded optic sensors: (**a**) polyimide-coated fibers; (**b**) acrylate-coated fibers.

Figure 5 shows the comparison of the calculated Young’s modulus for embedded and glued optic fibers. Figure 5a shows the results for the polyimide sensors (specimen #4 with glued sensor, specimen #5 with embedded sensor), while in Figure 5b the results of the acrylate sensors (specimen #1 and #2 for embedded sensors and specimen #6 for the glued sensor) are reported.

Regarding the polyimide sensors (Figure 5a), the Young’s modulus calculated with the embedded sensor has a slightly higher value (61.5 GPa) than that calculated from the strains measured by the sensor glued on the surface (58.7 GPa). The embedded sensor also shows very limited oscillations, which are also less regularly repeated than those obtained through the superficial sensor. Indeed, as the oscillations on the surface can be correlated to the fabric structure of the material, the measurement of the internal sensor is affected by both the upper and lower layers within which it is embedded, thus leading to nonperiodic oscillations and to limited amplitude variations. The lower mean value of the Young’s modulus obtained with the glued sensor can be due to free-surface effects, where the deformations are not restrained by proximal layers.

Regarding the acrylate sensors (Figure 5b), although both the sensors present the same U-shape, the Young’s modulus calculated with the embedded sensor has a slightly lower value (56.2 GPa) than that calculated from the strains measured by the sensor glued on the surface (61.7 GPa). This can be explained by considering that, differently from the glued sensor, in the embedded sensor, all the coating surface is in contact with the specimen, favoring the optic fiber deformation. Furthermore, the significant sensitivity of the sensor to the engaged length might also play a key role in the discrepancy between the embedded and the glued acrylate sensors. Indeed, the Young’s modulus calculated from the acrylate sensor embedded for 50 mm has a value of 133.3 GPa, which is similar to that obtained with the sensor glued for the same length.

### 3.2. Strain Gauge Results

**Figure 6:**
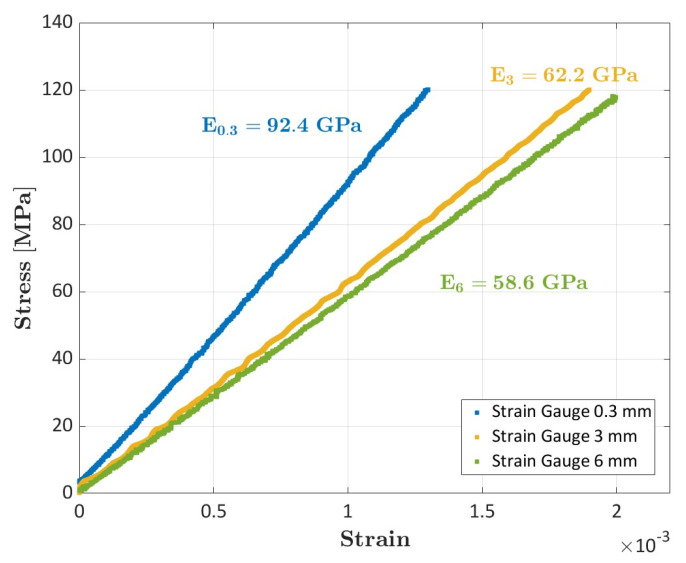
Stress-strain curves obtained with the strain gauges of dimensions 0.3 mm (specimen #3), 3 mm and 6 mm (specimen #1).

Figure 6 shows the stress-strain curves obtained with the strain gauges of dimensions 0.3 mm (specimen #3), 3 mm, and 6 mm (specimen #1).

**Figure 7:**
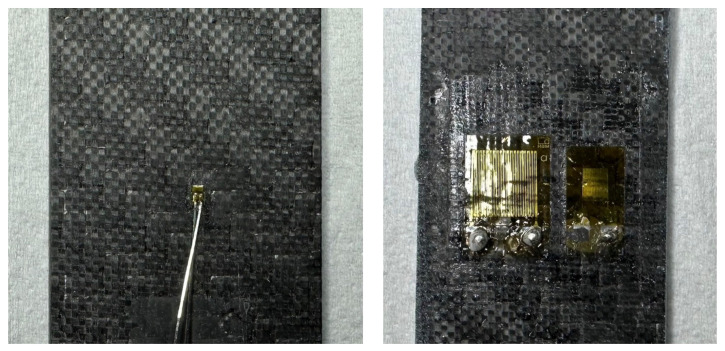
Magnification of the locations of the three strain gauges with respect to the material meso-scale structure.

The slope of the curve was clearly different between the three strain gauges and the Young’s modulus resulted equal to 92.4 GPa (0.3 mm strain gauge), 62.2 GPa (3 mm strain gauge), and 58.6 GPa (6 mm strain gauge). These consistent variations can be explained again by considering the nonhomogeneous strain field as result of the inhomogeneous meso-scale structure of the material. Figure 7 shows a magnification of the location of the three strain gauges.

As shown in the figure, the 0.3 mm strain gauge was mounted on a longitudinal tow, whose deformations were significantly smaller than average material deformations. The 0.3 mm strain gauge thus captures the local material behavior. According to Figure 7, the 3 mm strain gauge is mounted at the turn of longitudinal and transverse tows and thus only slightly overestimates the material Young’s modulus. Finally, the 6 mm strain gauge covers a wide area with respect to the material mesoscale structure and thus provides accurate results. These results suggest the use of strain gauges, whose dimensions are comparable with the material meso-structure size, can be critical as consistent overestimations or underestimations of the material properties can be obtained.

### 3.3. DIC Results

**Figure 8:**
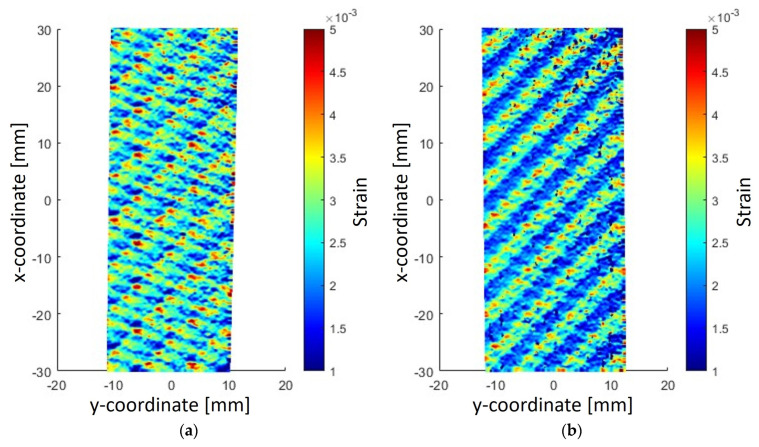
Full field strain map of the tensile test of the tested material: (**a**) specimen #1; (**b**) specimen #2.

Figure 8 shows the full field strain maps provided by the DIC for two different specimens in correspondence of the maximum load.

As shown, the strain field is inhomogeneous within the specimen, as result of the inhomogeneous meso-structure of the material constituted by longitudinal and transverse yarn with respect to the applied load. Indeed, transverse yarns, which can be seen as local unidirectional composite oriented at 90° with respect to the applied load, deform more than longitudinal tows. It is also possible to notice in the second specimen (Figure 8b) the woven pattern is easily recognizable through the bands of almost uniform deformation, while the strain field of the first specimen (Figure 8a) is more scattered. This can be attributed to the relative position of the material layers along the thickness direction: if the longitudinal and transverse tows of the two superficial layers perfectly superimpose, then the characteristic strain map is the result of almost uniform deformation bands [24].

**Figure 9:**
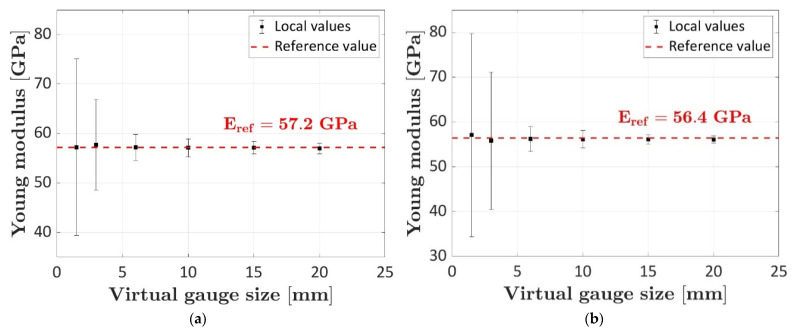
Computation of the Young modulus with virtual strain gauges of increasing size from the DIC strain field map: (**a**) computation on specimen #1; (**b**) computation on specimen #2.

To calculate the reference Young’s modulus, for each instant of the test, the strain field was averaged over the whole framed area. Furthermore, virtual strain gauges of dimensions 1.5 mm × 1.5 mm, 3.0 mm × 3.0 mm, 6.0 mm × 6.0 mm, 10.0 × 10.0 mm, 15.0 mm × 15.0 mm and 20.0 mm × 20.0 mm were randomly disposed on the framed area and the Young’s modulus was calculated by averaging the deformations measured by the DIC over the virtual strain gauges. Figure 9 reports the Young’s moduli calculated on the virtual strain gauges, the related uncertainty, computed by assuming a normal distribution and a 95% confidence interval, and the reference values of the Young’s moduli computed by averaging the deformations over the whole framed area.

The mean and the uncertainty were determined by repeating the calculation for 100 different random positions of each virtual strain gauge within the framed area. As shown in Figure 9, as 100 virtual strain gauges are considered for each size, the mean values are close or coincident with the reference Young’s modulus (57.2 GPa for specimen #1 and 56.4 GPa for specimen #2). However, as the size of the virtual strain gauge decreases, the uncertainty rapidly increases, thus suggesting small strain gauges, i.e., those whose dimensions are comparable with the material meso-structure size, must be adopted with particular attention. The considerable variation observed in smaller virtual strain gauges can be explained by applying the gauge primarily on matrix or fibers. It is also worth noting the confidence intervals of the 15 × 15 and 20 × 20 virtual strain gauges of specimen #2 are consistently smaller than those of the corresponding virtual strain gauges of specimen #1. This can be attributed to the uniform band shape of the strain field of specimen #2, as observed in Figure 8.

### 3.4. Comparison and Discussion

The Young’s moduli computed with the investigated techniques are summarized in Table 3.

For the sake of comparison, the superscripts refer to the specimen number, as indicated in Figure 2. Although the Young’s modulus can inherently vary between the specimens, as typical of composites, some general considerations can be drawn.

The optic fibers are strongly sensitive to the coating material and coating thickness. Results have shown the polyimide coating must be preferred over acrylate coatings for mechanical applications. The polyimide sensor provided accurate results, capturing the distinct deformations of the longitudinal and transverse tows of the material. In the polyimide sensor glued on the surface, the amplitude of the deformation oscillations caused by the material meso-structure was also comparable to that acquired by the DIC. According to the DIC, at 5 kN, the average strains of the longitudinal and transverse tows were 1500 and 2900 µε, respectively. In the polyimide sensors, the strain oscillated between 1750 and 2250 µε. In this regard, it is worth remarking the optic sensors have a strain gauge of 0.65 mm, i.e., the resolution of the optic sensors is much lower than that of the DIC. However, the embedment of polyimide optic sensor can be challenging as they are more prone to break during the curing cycle with respect to the acrylate-coated sensor. Furthermore, the polyimide coating favors micro-bending losses which can result in a too-weak light signal and affect in turn the strain measurement. Given their flexibility and refractive index properties, the acrylate-coated fibers are more reliable for embedment in structural applications, although their sensitivity to local material variations, such as those due to damage, can be critical. Furthermore, acrylate sensors represent a valid alternative for structural health monitoring only in case of long engaged length. Short acrylate sensors indeed provided incorrect Young’s moduli values, as reported in Table 3, being the acquired strains not consistent with those affecting the specimen. However, it is worth remarking even long acrylate sensors engaged within the specimen could not discriminate the deformations of the longitudinal and transverse bundles. Therefore, despite their manufacturability, the application of acrylate-coated fibers for the structural health monitoring of components made of carbon fiber woven composites can be critical.

Regarding the strain gauges, their size must be carefully selected in case of inhomogeneous composite materials whose strain field is nonuniform. For example, the Young’s modulus calculated with the 0.3 mm size strain gauge was more than 50% higher than the reference value. An analysis of the Young’s modulus variations calculated through virtual strain gauges of different sizes from the DIC acquisitions has also shown this issue. If the strain gauge size is smaller or comparable to the mesoscale structure of the material, a significant variability of the Young’s modulus is obtained. The DIC indeed provides the full field strain map and is therefore more suitable for inhomogeneous materials as the one considered here. However, it is worth remarking the strains measured on the surface can slightly differ from the internal ones. This has been shown with the embedded polyimide fiber, whose measurement was affected by both the upper and lower layers with oscillations of the measured strains significantly smaller than those measured on the specimen surface through the same sensor. Also, the comparison between the embedded and the glued polyimide fibers has shown the Young’s modulus calculated through the internal fiber was slightly higher than that calculated through the fiber mounted on the specimen surface, which is likely due to free-surface effects.

The three acquisition systems have also different costs and applications. The optic sensors and the Digital Image Correlation technique both require an important initial investment for the equipment (mainly investigator and cameras, respectively). However, the cost of a single test is almost null for both techniques, as the optic fibers can be cheaply manufactured by micro-welding a pigtail, the fiber, and the coreless terminal fiber, which can also be reused thereafter, and the Digital Image Correlation technique requires only to speckle the specimen. On the contrary, strain gauges do not require a consistent initial investment for the acquisition device, but the cost of each strain gauge is not negligible and mainly depends on its size. As for the setup time, micro-welding the optic sensors, mounting the strain gauges or spraying the specimen surface require similar operational time. However, the Digital Image Correlation also requires an initial calibration which must be repeated each time that the size of the framed area changes. Finally, it is worth remarking that, within the three techniques, only the optic sensors and the strain gauges are adopted for the structural health monitoring of in-service components, while the Digital Image Correlation is generally restricted to laboratory testing conditions.

Table 4 summarizes the strengths and weaknesses of the investigated strain measuring systems.

## 4. Conclusions

Given the inhomogeneous meso-structure of 2 × 2 twill woven composite, consisting of longitudinal and transverse yarns, strains caused by the application of a mechanical load are also inhomogeneous. Local damage mechanisms can thus affect the material, which demands for accurate information for the structural health state monitoring of these materials. Given the quantitative and distributed information, the embedment of optic sensors represents a promising solution, despite the challenging manufacturability of composite structures with embedded optic sensors. In this work, the effect of coating type, i.e., polyimide and acrylate, engaged length, i.e., 50 mm and 100 mm, and sensor position, i.e., embedded or glued on the specimen surface, on the strain acquisition have been investigated and compared to the strains acquired through strain gauges of different dimensions and through Digital Image Correlation (DIC). The specimens have been subjected to quasi-static tensile tests in the elastic field of the material. Optic sensors have been embedded in specimens made of four layers, in the middle of the laminate, i.e., between the second and the third layers. The optic fiber flaps coming off the specimen have been covered with releasing wax and a release paper film has been interposed between the optic fiber and the pre-pregs, to avoid the adhesion of the optic fiber to the specimen and prevent the fiber breakage during the cure cycle.

Results have shown the coating material strongly affects the strain measurement. Polyimide-coated sensors are able to capture the variations of the strain field, with oscillations of the deformation in agreement with the alternate presence of the longitudinal and transverse tows. The strain amplitudes measured by the polyimide sensors agreed with those acquired by the DIC system. Acrylate-coated sensors are instead strongly sensitive to the engaged length with short sensors, both embedded and glued, incorrectly measuring and underestimating the material strain. However, given their flexibility, acrylate sensors are easier to embed within the composite and less prone to breakage during the curing cycle with respect to polyimide sensors. The significant sensitivity of the acrylate sensors on the engaged length is caused by the coating material properties, mainly the Young’s modulus, and geometrical parameters, i.e., the thickness, of the coating, which suggests possible numerical approaches for its compensation. To the authors’ best knowledge, methodologies for modelling the acrylate sensors sensitivity to the engaged length have not yet been proposed in the literature and therefore represent possible future development. 

The inhomogeneous strain field of the material has been also observed with strain gauges of 0.3 mm, 3 mm, and 6 mm. The Young’s modulus calculated with the 0.3 mm size strain gauge was more than 50% higher than the reference value. An analysis of the Young’s modulus variations calculated from the DIC acquisitions through 100 virtual strain gauges of different sizes and randomly disposed over the framed area has also shown the uncertainty rapidly increases, as the size of the virtual strain gauge decreases. When the dimensions of strain gauges and tows of the fabric are comparable, strain acquisition can be critical.

This analysis has shown the importance of properly selecting the strain measuring system for structural health monitoring of composite materials and the results obtained could provide useful information for an appropriate choice. In particular, the use of acrylate-coated fibers for mechanical applications should be avoided, given their high sensitivity to the engaged length. For what specifically concerns woven fabric composites, the acrylate sensors were not able to distinguish the deformations of longitudinal and transverse bundles, thus limiting their application for the structural health monitoring of components made of this material. Furthermore, the use of small strain gauges on woven composite components should also be avoided. Finally, regarding the Digital Image Correlation technique, although it provides the most accurate and the densest information on the material deformation, its use is mainly limited to laboratory testing conditions.

## Figures and Tables

**Table 1:** Investigated strain acquisition systems and related addressed aspects.

Strain Acquisition System	Investigated Characteristics	
Optic fiber	i. Coating material Acrylate coating Polyimide coating	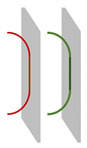
	ii. Sensor position Glued on the laminate Embedded within the laminate	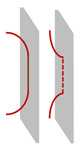
	iii. Engaged fiber length 50 mm 100 mm	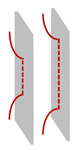
Strain gauge	i. 0.3 mm strain gauge size ii. 3.0 mm strain gauge size iii. 6.0 mm strain gauge size	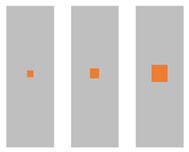
DIC	i. Full field strain map ii. Variable averaging area	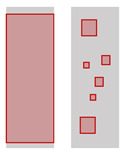

**Table 2:** Properties of the acrylate- and polyimide-coated optic fibers.

Property	G.657.A1 (Optokon)	SM1550P (Thorlabs)
Core material	Germanium doped silica	Germanium doped silica
Cladding material	Pure silica	Pure silica
Coating material	Dual layer of UV-cured acrylate	Polyimide
Core diameter (μm)	9	9
Cladding diameter (μm)	125	125
Coating diameter (μm)	242	145
Effective group index of refraction at 1550 nm	1.468	1.458
Maximum attenuation at 1550 nm (dB/km)	≤0.40	≤0.7

**Table 3:** Comparison of the Young modulus calculated with each strain acquisition system (superscripts numbers refer to the specimen number as indicated in Figure 2).

Strain Acquisition System		Young Modulus [GPa]
Optic fiber	Acrylate	133.32 (embedded for 50 mm)
		103.16 (glued for 50 mm)
		56.21 (embedded for 100 mm)
		61.76 (glued for 100 mm)
	Polyimide	61.55 (embedded for 50 mm)
		58.74 (glued for 50 mm)
Strain gauge		92.43 (0.3 mm strain gauge)
		62.21 (3 mm strain gauge)
		58.61 (6 mm strain gauge)
DIC		57.21
		56.42

**Table 4:** Summary of the strengths and weaknesses of the investigated strain measuring systems.

Measuring Device	Strengths	Weaknesses
Polyimide optic fiber	Capture the distinct deformations of the longitudinal and transverse tows (local measurement) Not sensitive to installation length. Surface and embedded strain measurement	Difficult installation: prone to break during the curing cycle. Prone to microbending losses
Acrylate optic fiber	The flexibility of the coating favors the installation. Surface and embedded strain measurement	Not sensitive to local material variations Sensitive to the sensor length.
Strain gauge	Easy installation with standard procedure	Only surface measurement. Local measurement. Resulting elastic property sensitive to gage position.
Digital Image Correlation	Full-field strain measurement	Only surface measurement High-dimension output file and long post-processing

## Data Availability

Data are available upon request.

## References

[1] Talreja R. (2016). Physical Modelling of Failure in Composites. Philos. Trans. R. Soc. A Math. Phys. Eng. Sci..

[2] Matthews F.L. Damage in Fibre-Reinforced Plastics; Its Nature, Consequences and Detection. Proceedings of the 3rd International Conference on Damage Assessment of Structures (DAMAS 99).

[3] Heslehurst R.B. (2014). Defects and Damage in Composite Materials and Structures.

[4] Schubel P.J., Crossley R.J., Boateng E.K.G., Hutchinson J.R. (2013). Review of Structural Health and Cure Monitoring Techniques for Large Wind Turbine Blades. Renew. Energy.

[5] Boursier Niutta C., Tridello A., Paolino D.S., Belingardi G. (2021). Residual Properties in Damaged Laminated Composites through Nondestructive Testing: A Review. Materials.

[6] Barrias A., Casas J.R., Villalba S. (2018). Embedded Distributed Optical Fiber Sensors in Reinforced Concrete Structures—A Case Study. Sensors.

[7] Sánchez D.M., Gresil M., Soutis C. (2015). Distributed Internal Strain Measurement during Composite Manufacturing Using Optical Fibre Sensors. Compos. Sci. Technol..

[8] Souza G., Tarpani J.R. (2020). Distributed Fiber Optics Sensing Applied to Laminated Composites: Embedding Process, Strain Field Monitoring with OBR and Fracture Mechanisms. J. Nondestr. Eval..

[9] Rufai O., Chandarana N., Gautam M., Potluri P., Gresil M. (2020). Cure Monitoring and Structural Health Monitoring of Composites Using Micro-Braided Distributed Optical Fibre. Compos. Struct..

[10] Kinet D., Mégret P., Goossen K.W., Qiu L., Heider D., Caucheteur C. (2014). Fiber Bragg Grating Sensors toward Structural Health Monitoring in Composite Materials: Challenges and Solutions. Sensors.

[11] Ramakrishnan M., Rajan G., Semenova Y., Farrell G. (2016). Overview of Fiber Optic Sensor Technologies for Strain/Temperature Sensing Applications in Composite Materials. Sensors.

[12] Qiu L., Goossen K., Heider D., O’Brien D., Wetzel E. (2011). Free-Space Input and Output Coupling to an Embedded Fiber Optic Strain Sensor: Dual-Ended Interrogation via Transmission. Opt. Eng..

[13] Luyckx G., Voet E., Lammens N., Degrieck J. (2011). Strain Measurements of Composite Laminates with Embedded Fibre Bragg Gratings: Criticism and Opportunities for Research. Sensors.

[14] Fernando G.F., Degamber B. (2006). Process Monitoring of Fibre Reinforced Composites Using Optical Fibre Sensors. Int. Mater. Rev..

[15] Seong Jang T., Lee J.J., Chun Lee D., Huh J. (1999). The Mechanical Behavior of Optical Fiber Sensor Embedded within the Composite Laminate. J. Mater. Sci..

[16] Padilla Michel Y., Lucci M., Casalboni M., Steglich P., Schrader S. Mechanical Characterisation of the Four Most Used Coating Materials for Optical Fibres. Proceedings of the 3rd International Conference on Photonics, Optics and Laser Technology.

[17] Weisbrich M., Holschemacher K. (2018). Comparison between Different Fiber Coatings and Adhesives on Steel Surfaces for Distributed Optical Strain Measurements Based on Rayleigh Backscattering. J. Sens. Sens. Syst..

[18] Weisbrich M., Holschemacher K., Bier T. (2020). Comparison of Different Fiber Coatings for Distributed Strain Measurement in Cementitious Matrices. J. Sens. Sens. Syst..

[19] Li E. Rayleigh Scattering Based Distributed Optical Fiber Sensing. Proceedings of the Applied Optics and Photonics.

[20] Thorlabs Coreless Termination Fiber. https://www.Thorlabs.Com/Newgrouppage9.Cfm?Objectgroup_id=7948.

[21] Thorlabs Single Mode Polyimide Coating Fiber. https://www.Thorlabs.Com/Thorproduct.Cfm?Partnumber=SM1550P.

[22] Sutton M.A., Orteu J., Schreir H.W. (2009). Image Correlation for Shape, Motion and Deformation Measurements.

[23] Ferrarese A., Boursier Niutta C., Ciampaglia A., Ciardiello R., Paolino D.S., Belingardi G. (2023). Experimental and Numerical Investigation of the Mesoscale Size Effect in Notched Woven Composites. Appl. Sci..

[24] Matveev M.Y., Long A.C., Brown L.P., Jones I.A. (2017). Effects of Layer Shift and Yarn Path Variability on Mechanical Properties of a Twill Weave Composite. J. Compos. Mater..

